# Annotations

**Published:** 1910-10-15

**Authors:** 


### Annotations.

### The Late Professor von Leyden.

In Ernst von Leyden Germany loses one of her "grand old men" of medicine—one of those indefatigable scientists who have worked unweariedly to promote the progress of knowledge, not merely in their own special branch but in cognate departments. As a clinician von Leyden reached a deservedly high position, and although he was one of the most retiring of men he became a popular man with the lay public, owing to his cheery outspokenness and his vigorous contempt for professional cant. The post-graduate student found in him a good friend, always willing to give counsel and to smooth over difficulties, and the International Committee, of which he was president, loses in him one of its most efficient members. He was an hereditary noble and the *doyen* of the Berlin faculty, and, indeed, his connection with Berlin was a feature in his whole career. He loved the city, and he loved the work that medical men were doing there, and it is no exaggeration to say that he himself contributed very largely to give the German capital that reputation for pre-eminence in medicine that it has enjoyed during the last five years. Although he retired from active work more than three years ago, his influence and personality still dominated the medical world at Berlin, and his loss will therefore create a gap which it will be very difficult to fill.

### The Portuguese Revolution.

From a medical point of view the disturbances in Portugal, which are now happily over and which have been accompanied with a singularly small percentage of casualties when we consider the environment in which they took place, present little of outstanding interest. The deposed King has not hitherto shown himself specially favourable to medicine, but he comes from a race that has always been keenly alive to the benefits which humanity has enjoyed from a cultivation of science in its various forms. His mother, we believe, holds a honorary degree in medicine, and has always taken a great interest in ambulance and first-aid work. On the Republican side the death, announced as the initial rumble of the gathering shock of revolution, of Dr. Bombarda, the well-known alienist and nerve specialist, of Lisbon, possesses somewhat more interest. Bombarda was not only a keen politician, almost fanatical in his aggressive Republicanism, but he was at the same time a widely read, courteous, and liberal-minded man of science, whose friendship and kindliness all those who attended the Medical Conference at Lisbon some years ago will recall with gratification. His death will be sincerely regretted by all, and through it the Republican party in Portugal loses a staunch adherent and a singularly able and generally respected leader. The change of *régime* is unlikely to affect the hospitals or the larger training-schools in Portugal, though the Royalist influence is said to be fairly strong at the University of Coimbra, where the King was until recently an undergraduate. The medical profession in Portugal has so far shown itself in close sympathy with the Republicans. The Lisbon and other local societies have adopted resolutions favouring the change and have allied themselves with the new *régime* as a matter of course. In the circumstances it is unlikely that there will arise any contingency in the near future which will enable members of the profession in this country to show their sympathy with their Portuguese *confrères* in some helpful and practical way.

### The Cholera Scare.

On Tuesday, the 4th inst., the evening papers reported, with entirely unnecessary headlines, the alleged discovery of a case of cholera at the Royal Free Hospital. The facts of the case are as follows: A fortnight ago the patient, a man aged twenty-seven years, was admitted into the male ward with a provisional diagnosis of typhoid. He was in a very much collapsed condition with a high temperature, and on the day following his admission he had diarrhœa, during which his temperature dropped to sub-normal. From that time until his death on Friday, the 7th inst., his condition was a "choleraic" one, and the clinical signs were analogous to those presented by a bad case of the condition which is still styled "cholera nostras." We need not remind our readers that this has absolutely nothing to do with cholera asiatica. It is a great pity that the nomenclature is still confusing, and that the old name "cholera nostras" has not been definitely discarded. A bacteriological examination was made, but nothing conclusive was found, although the existence of a bacillus—not necessarily a vibrio—was demonstrated. There is no reason whatever for the exaggerated statements which have appeared in some quarters in connection with the case. The so-called "endemic or English cholera," which is really a form of subacute enteritis, accompanied with profound collapse and with diarrhœa, is likely to be confounded with true cholera in times when the medical attendants are on the alert to detect any evidence of the latter disease. It is as well that this should be so, and although the confusion may on occasion give rise to some sensation, it is in the interests of the public that every suspicious case should be carefully scrutinised and investigated at a time when there is the remotest possibility of the real disease being in existence in the neighbourhood. There are several reasons against accepting the Royal Free case as one of true cholera. Into these, however, we need not go at the present moment: a full bacteriological investigation is being made, and doubtless in the course of the next few days the public will be informed whether the provisional diagnosis was the correct one, or whether the man really died of Asiatic cholera. Meanwhile it is worth while to point out that, apart from the dangers to which we recently alluded while discussing the chances of a cholera epidemic in this country, there need be no cause for anxiety or alarm.

